# Macronutrient Utilization After Short-term Fasting in Older and Younger Men and Women, a Pilot Study

**DOI:** 10.21203/rs.3.rs-5938311/v1

**Published:** 2025-02-18

**Authors:** San Wang, Rita Tsay, Danya Zhang, Daniel Cunha, Naomi Fukagawa

**Affiliations:** Massachusetts Institute of Technology; Massachusetts Institute of Technology; Boston University; Boston University; USDA ARS Beltsville

**Keywords:** fasting, aging, body composition, dual-energy X-ray absorptiometry, respiratory quotient, resting energy expenditure, substrate rate of appearance, substrate oxidation, lipolysis, stable isotope tracers

## Abstract

**Background/Objective:**

While older people are more prone than younger people to periods of involuntary fasting, systematic assessment of all the three major sources of macronutrient mobilization and oxidation in the same individual older participants during short-term periods of fasting has not been previously reported. Because aging is associated with many metabolic, hormonal, and body composition changes, older humans may have different kinetics of utilization of macronutrient stores during fasting than younger ones. This pilot study aimed to generate exploratory data to test this hypothesis.

**Methods/Subjects:**

We examined four groups of five participants each in this study, women and men, and older and younger subjects. We measured body composition by dual-energy X-ray absorptiometry (DEXA) and studied the effects of a 12-hour and a 36-hour fast on protein mobilization, lipolysis, and glucose output (substrate rates of appearance, using stable isotope tracers), as well as macronutrient oxidation.

**Results:**

The older participants had a greater percent body fat. Respiratory exchange ratios (RER) decreased with the longer fast. In a linear mixed model analysis of the metabolic data, age was not significant as a fixed effect when added to the model, except for glycerol rate of appearance and leucine oxidation rate.

**Conclusions:**

The effects of age and sex on mobilization and oxidation of macronutrient stores, as assessed with stable isotope tracers and indirect calorimetry, were small compared to the large overall effect of a 36-hour fast, suggesting that the macronutrient metabolic switching of older people with fasting is similar to that of younger people.

## INTRODUCTION

The metabolic transitions with short-term fasting in older people are of particular interest in clinical nutrition, since periods of minimal food intake are common occurrences, due to illness, dementia, depression or social isolation (1, 2, 3, 4). The physiologic and metabolic adaptations during fasting are complex (5). These changes lead to increased hepatic glycogenolysis and gluconeogenesis to maintain blood glucose levels for central nervous system (CNS) function (6). They also stimulate adipocyte lipolysis and beta oxidation of fatty acids in the liver for formation of ketone bodies such as betahydroxybutyrate to eventually replace glucose as a CNS energy source (7), as well as skeletal muscle proteolysis to provide amino acids for hepatic and renal gluconeogenesis (8). Initially, with fasting, there is an increase in resting energy expenditure (REE) due to the energy needed for the adaptations of gluconeogenesis, ketogenesis and triglyceride-fatty acid, protein-amino acid, and acetyl-CoA-ketone body recycling (8, 9). A rise in sympathetic nervous system activity also increases REE, though as fasting is prolonged for more than two days, REE decreases (8). There is also a transient increase in protein and free amino acid oxidation (particularly glutamine) and higher urine nitrogen excretion (5). All these metabolic and hormonal responses to fasting could be affected by age. Furthermore, body composition changes with age, with decreased skeletal muscle mass and increased visceral fat (1 0), changes that could also have effects on the metabolic and physiologic responses to short-term fasting.

Although there have been several previous reports of studies of specific macronutrient metabolism during overnight and short term fasting comparing older to younger men or older to younger women (11, 12, 13, 14, 15), we are not aware of any studies directly comparing the effects of short term fasting of up to 36 hours in all four groups of people using identical protocols for the three major macronutrient substrates. Here, we report a pilot study to explore potential differences with both age and sex in the metabolic response to short-term fasting, as manifested by macronutrient utilization. We measured body composition by dual-energy X-ray absorptiometry (DEXA) and studied the effects of a 12 hour (overnight) and a 36 hour fast on protein mobilization, lipolysis, and glucose output (i.e., substrate rates of appearance), as well as protein, fat, and carbohydrate oxidation (i.e., substrate oxidation). Thus, we could make an exploratory assessment of all the major sources of macronutrient mobilization and oxidation in healthy older and younger men and women subjects following brief periods of fasting of up to 36 hours.

## PARTICIPANTS AND METHODS

### Participants.

Ten older (60–81 years) and ten younger (18–28 years) participants were studied, with 5 men and 5 women in each age group, each person undergoing both a 12 hour and a 36 hour fast. Participants were healthy volunteers with BMI ^1^s below 30. Exclusion criteria included diabetes mellitus, cardiac, renal, or hepatic disease. Older participants with hypertension but with BP < 140/80 on antihypertensive medications were included, as was one older participant on Synthroid with a normal TSH. Smokers were excluded. Younger women were studied within 7 days of cessation of their last menstrual period, and had a urine human chorionic gonadotropin test to exclude pregnancy just prior to each infusion.

A detailed description of the participants’ stable isotope infusion protocols for the 12 hour and 36 hour fasts, collection and analysis of samples, indirect calorimetry, and calculations is given in the Supplemental Material in the Methods section, as well as a description of the use of dual energy X-ray absorptiometry to assess the participants’ body composition.

### Statistical Analysis.

Box plots of the metabolic data displayed large interquartile ranges (IQR) and frequent outliers (> 2.5 IQR above the 3rd or below the 1 st quartiles) for each of the four groups of participants. For ANOVA analysis of the data to be valid, one must verify that the assumptions of independent observations, equal variances, and normal distributions are valid. However, QQ plots showed frequent deviations from normality when the data from the 12 hour and 36 hour fasts were considered separately. Rather than using a repeated measures ANOVA to analyze the data, and in order to maximize the use of the data collected, we constructed a mixed effects linear model combining the data from the 12 hour and the 36 hour fasts and controlled for intrasubject correlation with a random intercept. We were thus able to use all 40 observations for each dependent metabolic variable of interest to infer the effect of age and sex on the variable.

Visual inspection of plots of residuals versus fitted values did not show any obvious deviations from homoscedasticity. QQ plots showed some deviations from normality, but regression models are fairly robust to deviations from normality. Addition of random slopes for each participant to the analysis did not affect conclusions from the analysis. Scatter plots of dependent metabolic variables for substrate rates of appearance and oxidation did not show any clear correlations with activity level, BMI, fat mass, fat mass/height squared, arm and leg mass, and arm and leg mass/height squared, so these were not included as fixed effects in our model. Because of the small number of participants per age sex category, we did not remove outliers. Our final mixed effects linear model had age and sex and an indicator variable for 12 or 36 hours fast as the fixed variables, and a random intercept for each participant. Analysis was done with the Ime4 program in R (22). Lme4 gives results as intercepts and effects of the fixed effects on the intercept. In order to calculate P-values, we used a Chi Squared log likelihood ratio test (22) to compare the model with and without the factor of interest (length of fast (12 hours/36 hours), age and sex, as well as the interaction of age and sex). We present our results both in the Ime4 summary format, and as P values.

Data in the tables are given as means ± SD. Because of risk of increased Type I errors due to multiple comparisons of the metabolic data with four categories of participants, we used P < 0.01 as our cutoff for statistical significance.

## RESULTS

### Body Composition (Table 1)

The mean weight of the men was greater than the women in both age groups. The mean percent fat was greater in women than men, and greater in the older participants. Mean total lean mass, as measured by DEXA, was 50% greater in men than women, and was less with age. Creatinine excretion (a more direct measure of skeletal muscle mass) was also lower in the older participants than the younger (30% lower in both older men and older women, Table 1). There was no significant difference in body mass index (BMI) between the younger and older participants, or between men and women.

### Substrate Plasma Levels (Supplementary Table Sl).

Plasma glucose levels after an overnight (1 2 hour) fast were significantly higher in the older participants than in the younger ones, and decreased by 0.5–1 .0 mmol in all participants with the 36-hour fast. Leucine levels were similar in the younger and older participants after a 12-hour fast, but almost doubled after a 36-hour fast in the younger participants. In the older participants, plasma leucine levels increased only 20% after a 36-hour fast, compared to their levels after a 12 hour fast.

### Metabolic Data

**Figure 1:**
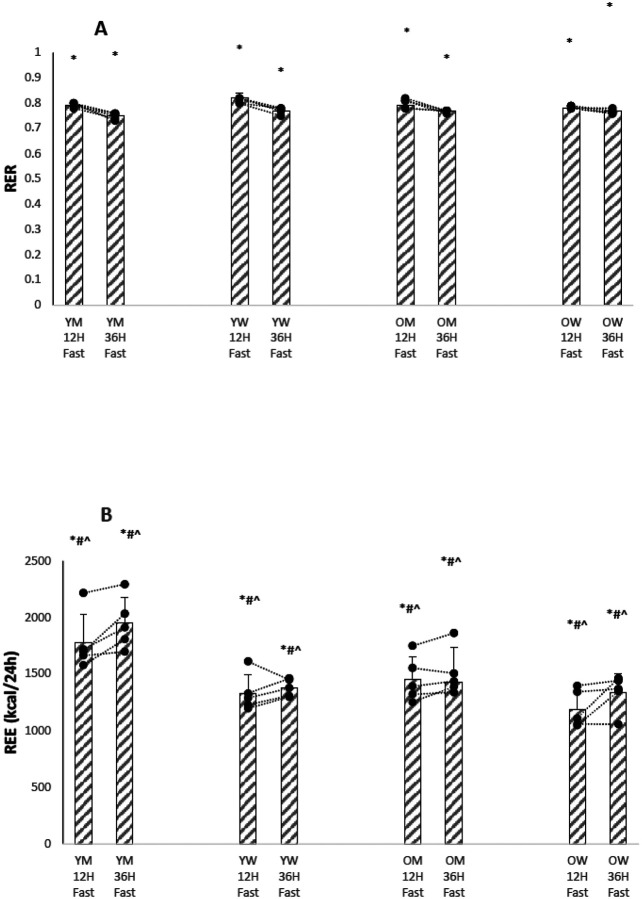
(A). RER after 12 and 36 Hours of Fasting for Individual Participants. (YM younger men, YW younger women, 0M older men, OW older women) * P<0.001 12h Fast vs. 36h Fast (B). REE after 12 and 36 Hours of Fasting for Individual Participants. (YM younger men, YW younger women, 0M older men, OW older women) * P<0.001 1 211 Fast vs. 36h Fast # P<0.001 Younger vs. Older ^ P<0.001 Men vs. Women

Figure 1 (A,B)shows bar graphs of means, standard deviations (SD), and individual participant values for RER and REE in the four groups after a 12 hour and after a 36 hour fast, as well as the points of the individual five participants in each group, and SupplementaryTable S2 summarizes the results. As expected, the RER values fell with the more prolonged fast, indicating a shift to fat oxidation from glucose oxidation. There was no effect of age or sex on the magnitude of this shift. REE increased withe the more prolonged fast, and age and sex both affected REE values at the P<0.001 level.

**Figure 2:**
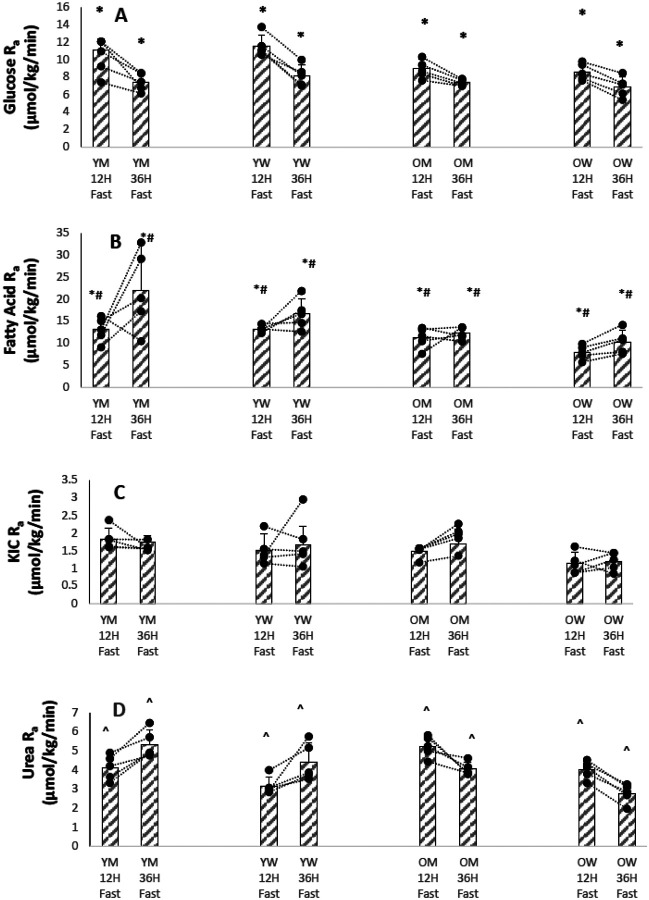
(A). Glucose Rate of Appearance (YM younger men, YW younger women, 0M older men, OW older women) * P<0.001 12h Fast vs. 36h Fast (B). Fatty Acid Rate of Appearance after 12 and 36 Hours of Fasting for Individual Participants. (YM younger men, YW younger women, 0M older men, OW older women) * P<0.01 12h Fast vs. 36h Fast # P<0.01 Younger vs. Older (C). KIC Rate of Appearance after 12 and 36 Hours of Fasting for Individual Participants. (YM younger men, YW younger women, 0M older men, OW older women) (D). Urea Rate of Appearance after 12 and 36 Hours of Fasting for Individual Participants. (YM younger men, YW younger women, 0M older men, OW older women) ^ P<0.001 Men vs. Women

Figure 2(A,B,C,D) shows bar graphs of means, SD , and individual participant values of substrate and urea rates of appearance for the four groups after a 12 hour and after a 36 hour fast, and Supplementary Table S3 summarizes the results. With a 36-hour fast, the R_a_ of glucose fell in all participants compared to the values after a 12-hour fast.

Fatty acid R_a_, a measure of triacylglyceride breakdown, was significantly lower in the older participants. Even though glucose R_a_ decreased with a 36-hour fast, overall resting energy expenditure did not, and was just as high, if not higher, than after a 12-hour fast (Figure 1 B). The energy deficit caused by decreased glucose R_a_ with the longer fast was largely made up by increased triacylglyceride breakdown, as reflected by an increase in fatty acid R_a_ (Figure 2B) and a decrease in RER (Figure 1A).

Amino acid availability, as reflected by R_a_ of KIC, was not affected by age or sex (Figure 2C). However, urea Ra, a measure of overall protein catabolism, was lower in women (Figure 2D), perhaps because of their lower total lean mass.

**Figure 3:**
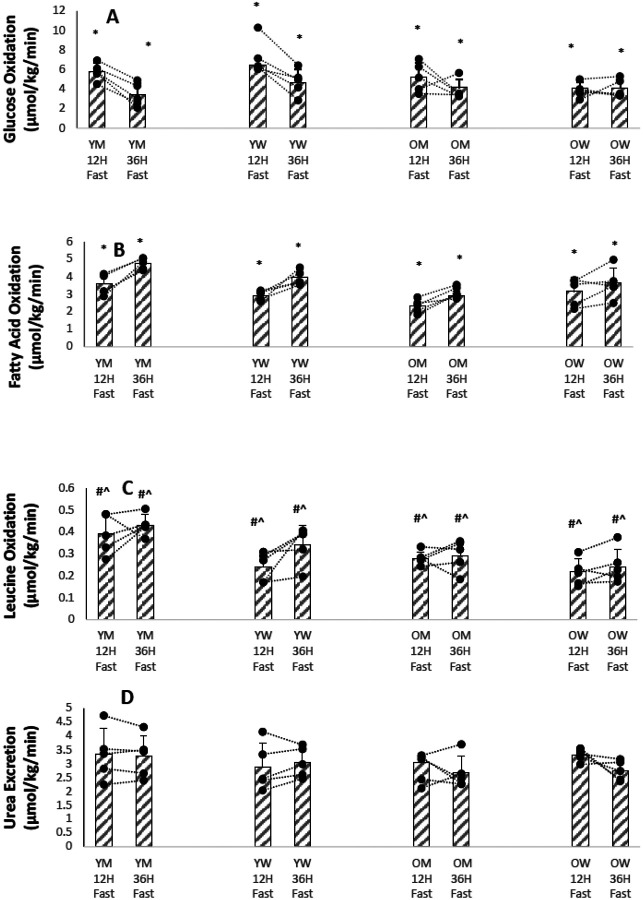
(A). Glucose Oxidation from REE after 12 and 36 Hours of Fasting for Individual Participants. (YM younger men, YW younger women, 0M older men, OW older women) * P<0.001 12h Fast vs. 36h Fast (B). Fatty Acid Oxidation from REE REE after 12 and 36 Hours of Fasting for Individual Participants. (YM younger men, YW younger women, 0M older men, OW older women) * P<0.001 1 211 Fast vs. 36h Fast (C). Leucine Oxidation from ^13^C0_2_ Excretion after 12 and 36 Hours of Fasting for Individual Participants. (YM younger men, YW younger women, 0M older men, OW older women) # P<0.01 Younger vs. Older ^ P<0.01 Men vs. Women (D). Urea Excretion after 12 and 36 Hours of Fasting for Individual Participants. (YM younger men, YW younger women, 0M older men, OW older women)

Figure 3(A,B,C,D) shows bar graphs of means, SD, and individual participant values for substrate oxidation rates and urea excretion rates for the four groups of participants after a 12 hour and after a 36 hour fast and Supplementary Table S4 summarizes the results. The differences between means of glucose and fatty oxidation after a 12 hour fast and after a 36 hour fast were significant at P < 0.001 . Age and sex had no significant effect on the means. However, differences between leucine oxidation between younger and older participants and between men and women were significant at P < 0.01 . The rates of appearances of the three substrates examined (glucose, fatty acids, KIC) in general paralleled that of the oxidation of their respective fuel categories (glucose, fatty acids, and leucine (Figures 2 and 3 and Supplementary Tables S3 and S4)).

**Figure 4:**
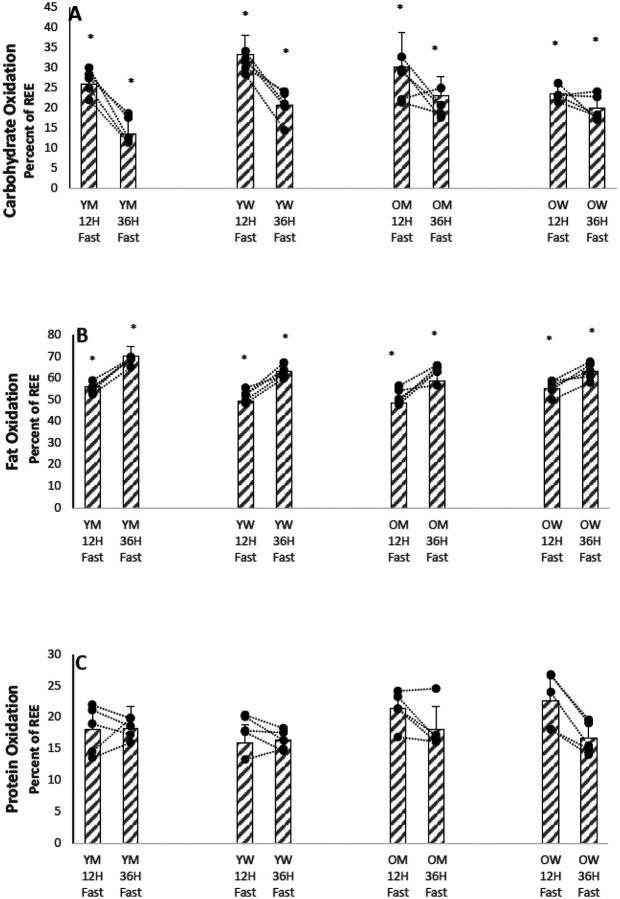
(A). Percent REE from Carbohydrate after 12 and 36 Hours of Fasting for Individual Participants. (YM younger men, YW younger women, 0M older men, OW older women) * P < 0.001 12h Fast vs. 36h Fast (B). Percent REE from Fat after 12 and 36 Hours of Fasting for Individual Participants. (YM younger men, YW younger women, 0M older men, OW older women) * P<0.001 12h Fast vs. 36h Fast (C). Percent REE from Protein after 12 and 36 Hours of Fasting for Individual Participants. (YM younger men, YW younger women, 0M older men, OW older women)

Figure 4(A,B,C)shows bar graphs of means, SD, and individual participant values for the calculated percent of the REE contributed by oxidation of the three main metabolic macronutrients (carbohydrates, fat and protein), and Supplementary Table S5 summarizes the results. Fat oxidation supplied 50–60% of the total REE after an overnight fast in all groups of participants, and glucose oxidation supplied 20–30% of REE. Protein oxidation (extrapolated from urea excretion) contributed a surprisingly high 16–18% of the total REE of younger participants, and even higher amounts (21–23%) in the older participants. As fasting was prolonged to 36 hours from 12 hours, the percent contribution of glucose oxidation to total REE markedly decreased and the contribution of fat oxidation increased in all groups of participants. The differences between values after a 12 hour fast and after a 36 hour fast in the percent of the REE contributed by carbohydrate and the percent of the REE contributed by fat were both significant at P < 0.001 . Age and sex had no significant effect on the means, and the percent of the REE contributed by protein was not significantly affected by age, sex, or the length of the fast, and remained in the 16–23% range.

Figures 1–4 show that there is marked heterogeneity in the responses of the individual participants to fasting, with much overlap between the four groups. Supplementary Tables 2–5 show summaries of the statistical analyses as P < 0.01 or P < 0.001 from the mixed linear models of the data. If no P values are given, the analyses gave P values > 0.01 . Supplementary TablesS6-S9 show the actual results from the statistical analyses of the data using a mixed effects linear models of the metabolic variables measured. These models calculate the mean value of the metabolic dependent variable of interest after a 12 hour fast as a baseline intercept value and the changes in the intercept value due to the fixed effects of age, sex, and a 36 hour fast. For the linear mixed model, age was treated as a continuous variable, so the change in intercept value must be multiplied by the participant’s age. The tables also show the P-values (calculated from a Chi-Squared likelihood ratio test) that the baseline 12 hour fast value of the metabolic variable was different in the linear mixed model if the fixed effect (age, sex, or 36 hour fast) was added as a factor to the model. In general, the results show that the fixed effects of age and sex on a metabolic variable were small compared to the fixed effect of a 36 hour fast on the baseline 12 hour fast value, and for most metabolic variables did not affect the linear mixed model at a P<0.01 level; furthermore, there was usually no interaction between age and sex. For fatty acid rate of appearance and leucine oxidation, age was significant as a fixed effect on the linear mixed model, in both cases lowering the mean value.

## DISCUSSION

In this pilot study, we sought to evaluate the effects of age and sex on macronutrient substrate appearance (mobilization) and oxidation (utilization) in humans after short periods of fasting (12 hours and 36 hours).

The rate of appearance of glucose was 1.5–2 times the rate of its oxidation (Figs. 2 and 3). How this additional glucose is disposed of other than by oxidation is unknown. Some of the non-oxidative disposal of glucose could be in production of glycerol-3-phosphate for free fatty acid re-esterification (7). Some glucose is also disposed of by conversion to lactate through the Cori cycle. Much of the glucose R_a_ during fasting is supplied by gluconeogenesis. Rothman et al. have found that most of the total glucose production in humans is from gluconeogenesis rather than glycogenolysis (6). Even early in the post-absorptive period, when liver glycogen stores are maximal, gluconeogenesis constitutes 50% of hepatic glucose production, and this increases to 82% at 36 hours (6).

Protein oxidation, calculated from urinary urea nitrogen excretion, was fairly constant in all four groups of participants, when calculated as grams oxidized per kilogram of body weight. When protein oxidation was calculated from leucine oxidation, the younger men had the highest rates of protein oxidation, and these were similar to those calculated from urinary urea excretion. For the other three groups of participants, protein oxidation calculated from leucine oxidation was as much as 50% lower than values calculated from urea excretion. The reasons for this discrepancy between these two measurements of protein oxidation are unclear, but could be due to preferential oxidation of nonessential amino acids, such as alanine and glycine, in the women and older men. Although there is a clear loss of skeletal muscle mass with aging, using phenylalanine, an essential amino acid that is not oxidized in muscle, Volpi et al found that older men had higher muscle protein synthesis rates and higher breakdown rates, so that they showed no significant net age-related difference in basal muscle protein turnover rates compared to younger men (23). However, Smith et al. found that older women had higher rates of muscle protein fractional synthesis rates compared to younger women (24).

Urea R_a_, another measure of protein oxidation was higher in men than in women (Fig. 2D, P < 0.01), even though urinary urea excretion was not different between the sexes. The most likely explanation for this discrepancy is that men have higher rates of intestinal urea hydrolysis than women (25).

Most other studies that have examined the effects of short periods of fasting on substrate R_a_ and oxidation have generally been confined to younger participants (11, 13, 14, 15, 26, 27, 28, 29). All of these independent studies found values for their metabolic variables comparable to the values reported here, which suggests that the results of this pilot study would be reproducible if a larger number of participants had been studied.

A limitation of this study were the large standard deviations (wide confidence intervals) of the results, making it harder to draw definitive conclusions in comparisons between the older and younger participants and men and women. The variations are probably a reflection of the genetic heterogeneity of humans. One should also note that one cannot definitively rule out a type 2 error even if the present study shows no significant differences between younger and older participants or men and women. Given enough participants, one may indeed find a statistically significant difference in age or sex, but the difference might be so small as to be of little clinical importance.

Many studies have shown health benefits from intermittent fasting, where the metabolic switching from glucose to fatty acids as the main source of energy leads to an adaptive systemic and cellular responses that reset intermediary metabolism, increase oxidative stress resistance, decrease inflammation, and enhance cellular maintenance and repair pathways (reviewed in 30,31). Prolonged periods of fasting were likely frequent occurrences for humans during their evolution, and the metabolic stress of food deprivation would have been present for young and old and both sexes equally. Thus, it may not be surprising that the metabolic adaptations to fasting are generally similar for age and gender.

In conclusion, despite differences in body composition between older and younger people and women and men, differences during short term fasting in the metabolic parameters examined were mostly small. Protein mobilization and utilization remained as high in our older participants as in the younger ones, despite a large difference in lean mass in the older participants and protein oxidation made as large a contribution to total REE in the older participants as in the younger ones. This may make older people more prone to loss of skeletal muscle mass with the poor intake seen during hospitalizations, illnesses, and other periods of stress, and suggests that clinicians should prioritize adequate protein intake in their older patients to prevent depletion of body protein stores (32, 33). The similarity of macronutrient kinetics and utilization in younger and older participants during short term fasting suggests that older people might also see health benefits from the cellular and systemic responses induced by fasting (30).

## Figures and Tables

**TABLE 1:** Weight and Body Composition of Participants (Means±SD)

	Younger Men	Younger Women	Older Men	Older Women
	N=5	N=5	N=5	N=5
Age (y)	24.5±5.9*	22.6±4.8*	71.8±10.4*	75.0±3.5*
Weight (kg)	77.2±10.8^	59.9±9.5^	81.1±9.9^	63.8±8.0^
% Fat	14.5±4.7*^	25.1±7.2*^	24.4±4.8*^	33.9±5.0*^
Arm and Leg Lean Mass (kg)	26.9±4.7^^#^	17.0±2.2^^#^	24.2±3.7^^#^	14.7±1.8^^#^
Trunk Lean Mass (kg)	29.3±4.3^^#^	21.4±2.3^^#^	30.0±2.3^^#^	21.3±1.7^^#^
Total Lean Mass (kg)	57.0±7.8^^#^	38.7±4.2^^#^	56.2±5.1^^#^	36.0±3.2^^#^
BMI (kg/m^2^)	23.8±2.6	22.4±2.9	27.8±2.2	25.4±4.0
Creatinine Excretion (mg/kg/24h)	27.3±4.8*	23.3±4.9*	19.4±3.1*	16.3±1.8*

* P < 0.01 Young vs. Old

^ P < 0.01 Men VS. Women

# P < 0.01 YM vs. YW, YM vs. OW, 0M vs. YW, OM vs. OW

## Data Availability

Data described in the manuscript will be made available on request.
